# Adaptation of an evidence-based home cardiac rehabilitation programme for people with coronary heart disease in Bangladesh

**DOI:** 10.1186/s12913-026-14056-6

**Published:** 2026-03-20

**Authors:** Jamal Uddin, Mithila Faruque, Saidur Rahman Mashreky, Yousra Siddiqueea, Chaudhury Meshkat Ahmed, Md. Fakhrul Islam Khaled, Md. Rezaul Karim, Carolyn Deighan, Louise Taylor, Hasnain Dalal, Rod S. Taylor

**Affiliations:** 1 MRC/CSO Social and Public Health Sciences Unit, Institute of Health and Wellbeing, University of Glasgow, Glasgow, UK; 2 Physiotherapy and Cardiac Rehabilitation Unit, Department of Cardiac Surgery, Ibrahim Cardiac Hospital and Research Institute, Dhaka, Bangladesh; 3 Department of Non-Communicable Diseases (NCD), Faculty of Public Health, Bangladesh University of Health Sciences, Dhaka, Bangladesh; 4 Department of Public Health, North South University, Dhaka, Bangladesh; 5 University Cardiac Centre, Department of Cardiology, Bangabandhu Sheikh Mujib Medical University, Dhaka, Bangladesh; 6 Department of Cardiology, Ibrahim Cardiac Hospital and Research Institute, Dhaka, Bangladesh; 7 NHS Lothian, Heart Manual Department, Astley Ainslie Hospital, Edinburgh, UK; 8 Primary Care Research Group, Department of Health and Community Sciences, University of Exeter Medical School, Exeter, UK; 9 MRC/CSO Social and Public Health Sciences Unit & Robertson Centre for Biostatistics, Institute of Health and Wellbeing, University of Glasgow, Glasgow, UK

**Keywords:** Home-based cardiac rehabilitation, Adaptation, Heart manual, Post-myocardial infarction, Revascularisation

## Abstract

**Background:**

Cardiac rehabilitation (CR) is a clinically proven cost-effective intervention and reduces the risk of hospital readmissions and recurrent cardiac events and improves exercise capacity and health-related quality of life in people. However, access to CR in low and middle-income countries remains scarce and a high unmet need. The purpose of this study was to adapt a home-based CR programme (‘the Heart Manual’) originally developed and evaluated in the United Kingdom (UK) for the people with coronary heart disease (CHD) in Bangladesh.

**Methods:**

Guided by the 2021 ADAPT methodological framework, a multiphase approach to intervention adaptation was undertaken that included mapping of the Bangladeshi healthcare context and in-depth consultation meetings and workshops with key stakeholders (patients and their families, healthcare professionals, and health service providers).

**Results:**

In addition to language translation (English to Bangla) and cultural adaptation, the UK Heart Manual content and structure were modified whilst retaining its core interventional components and principles. The resulting Bangla Heart Manual comprises of three main adaptions: (1) combination of the UK Heart Manual post-myocardial infarction and revascularisation manuals into a single document; (2) development of a short version for easy reference for patients; and (3) creation of audiovisual and animated versions of materials to promote patient accessibility.

**Conclusion:**

Using an evidence-based approach, the UK Heart Manual programme was contextually and culturally adapted for people with CHD in the Bangladesh healthcare setting. The Bangla Heart Manual provides the opportunity for access to home-based CR in a low resource setting. Research is needed to confirm the feasibility and acceptability of the delivery of the adapted Bangla Heart Manual for healthcare practitioners, patients, and their families.

**Supplementary Information:**

The online version contains supplementary material available at 10.1186/s12913-026-14056-6.

## Introduction

Coronary heart disease (CHD) is the single major cause of global disability and death [1, 2]. By 2030, more than 80% of the impact of heart disease will occur in low- and middle-income countries (LMICs) [3]. In the South Asian region, heart disease often affects people of working age resulting in major socio-economic burden [4].

Cardiac rehabilitation (CR) is a clinically proven cost-effective intervention. CR has been shown to reduce the risk of hospital readmissions and recurrent cardiac events and improve exercise capacity and health-related quality of life in people with CHD and heart failure [5–8]. CR is a multifaceted secondary prevention intervention, that typically involves structured exercise based on an exercise prescription, health education, lifestyle modification and psychological support [8]. Despite CR being is a level I and grade A recommendation by both American Heart Association/American College of Cardiology, the European Society of Cardiology, and other international guidelines [9–11], participation rates of CR remain stubbornly low. The World Health Organisation has identified CR as a priority in its Global Action Plan for the Prevention and Control of Noncommunicable Diseases [9]. Access to CR is particularly poor in LMICs where the burden of CHD is high and increasing [10, 11].

CR has traditionally been delivered in a centre or hospital-based setting supervised by healthcare professionals [5]. However, centre-based CR poses potential barriers to patient attendance including the time, inconvenience and cost of travelling, absence from employment, and challenges in involving caregivers [5, 12] Home-based programmes provide an alternative affordable and scalable model of CR for LMICs that can overcome many of these barriers. The COVID-19 pandemic has provided a unique opportunity for healthcare systems across the globe to fast track the development and delivery of home and digitally supported remote models of care [13–15]. Randomised trials have shown that home and digitally supported models of CR can be as effective and safe as centre-based programmes [16]. Nevertheless, many of these trials have been conducted in high income countries which may lack generalisability and applicability to the LMIC setting [8].

Adaptation is defined as an ‘intentional modification(s) of an evidence-informed intervention, to achieve a better fit between an intervention and a new context [17–19]. Adapting existing evidence-based interventions can be a more efficient effective strategy than creating new interventions.

This study aimed to undertake adaptation of the Heart Manual home-based CR programme originally developed and evaluated in the United Kingdom (UK) to develop the ‘Bangla Heart Manual’ for use by people with CHD and their families in Bangladesh.

## Methods

### Study design

This study was designed in accordance with the 2021 ADAPT guidance [20] that proposes four steps to the adaptation of an intervention: (1) assessment of rationale for intervention and intervention and context fit, (2) plan and undertake adaptations, (3) piloting and evaluation of adapted intervention, and (4) implementation of the adapted intervention. This paper reports the first two of these four steps (i.e., adaptation of the UK Heart Manual for people with CHD in Bangladesh) and will inform future planned piloting and implementation of this adapted intervention. In addition to language (English to Bangla) translation, we anticipated that adaptation of the UK Heart Manual would need to address potential contextual differences between countries, including the needs of Bangladeshi people living with CHD and their caregivers and the Bangladeshi health and social care systems (see e-Table 1).

The study was undertaken between 1st May 2023 and 29th February 2024. The protocol for the study was pre-registered on the Open Science Framework (https://osf.io/tuakh/).

## Procedure

**Fig. 1:**
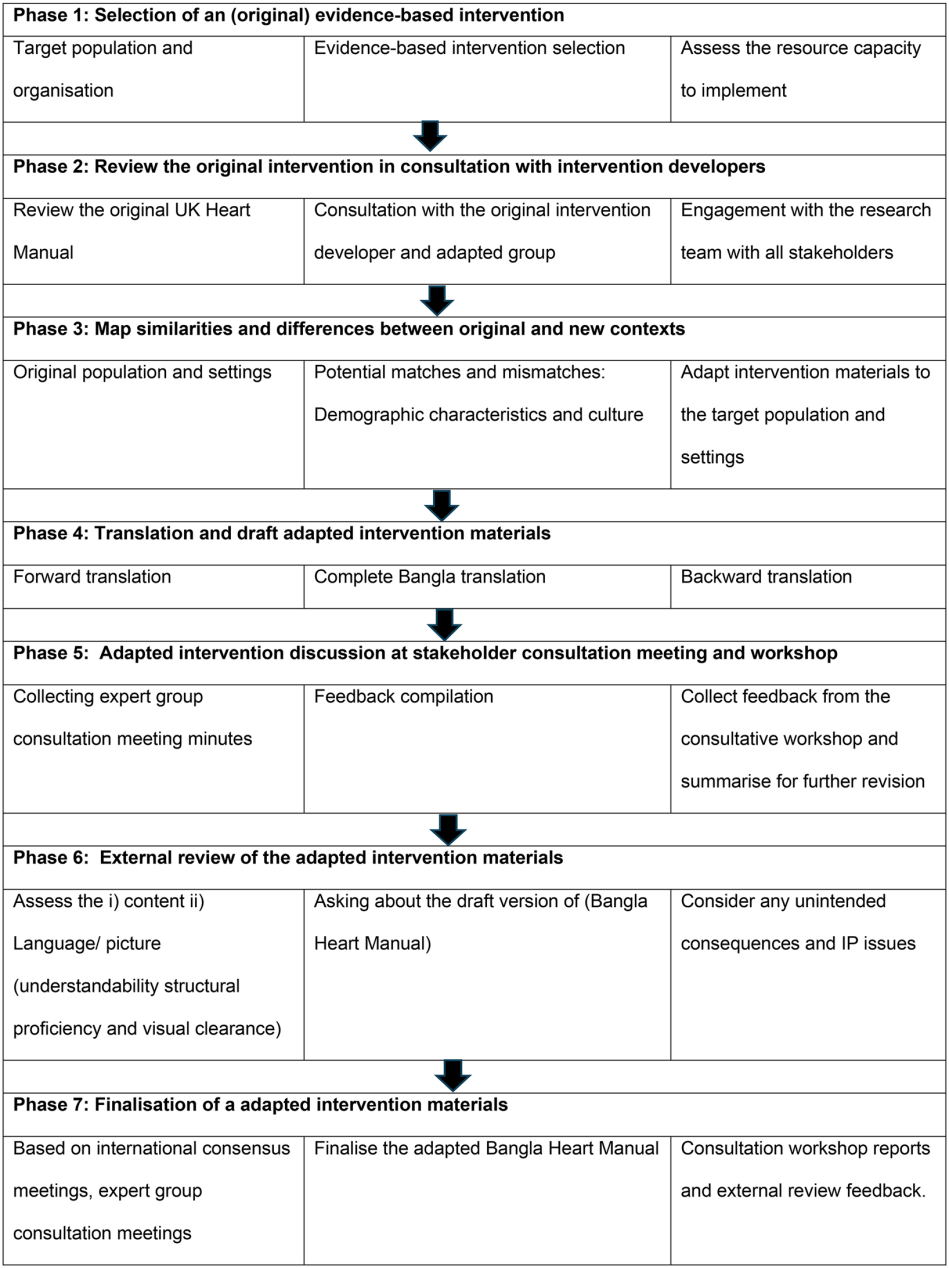
Seven phase adaptation procedure

The process for adapting public health evidence-based interventions [21, 22] and 2021 ADAPT guidance [20] were combined into a seven-phase procedure for purposes of this study (see Fig. 1). This procedure is summarised below (see e-Appendix 1 for further details).

We purposively identified study participants/stakeholders who were relevant to the adaptation process. We first held an international expert group consultation meeting that include from the (Lothian Health Board) of the original UK Heart Manual developer, UK users of the Heart Manual, and Bangladeshi healthcare professionals. Following this meeting, two national meetings were held: (1) an expert group consultation meeting was held on 10th October 2023 including consultant cardiologist, cardiac surgeon, public health expert, senior physiotherapist, psychiatrist, senior staff nurse, and nutritionist, followed by (2) a consultative workshop was held on 10th January 2024 with stakeholders likely to involved in the delivery and receipt of the Heart Manual in Bangladesh. This included a medical officer of cardiology, medical officer of cardiac surgery, physiotherapist, cardiac staff nurse, medical technologist, patient counsellor, dietitian, patients with CHD (post myocardial infraction and revascularisation), and their caregivers. We collated all feedback from consultative workshops attendees based on their completed semi-structured questionnaires, see (see e-Appendix 2). Following an expert group consultation meeting and a consultative workshop with different healthcare professional experts, service providers, patients and their caregivers to check the Bangla Heart Manual understandability and deliverability. We discussed in these two national consultation meetings for the adaptation and appropriateness of the content of the UK Heart Manual, the sequence of material content, use of language/pictures, and understandability of materials (see e-Appendix 3. A final consensus meeting was held to finalise all the training materials for the patients and facilitator training materials and to confirm the intervention delivery approach and a summary of the participants in the process stakeholders is shown in Table 1.

### Data analysis

Consultation meetings and workshops were audio recorded which was the analysed to produce draft meeting minutes that were approved by participants. The Bangla Heart Manual and materials were modified based on participant feedback suggestions. This data analysis process was coordinated by two researchers and supported by a third researcher.

## Results

### Initial review of UK heart manual intervention

The initial review of the UK Heart Manual material identified four key areas for adaptation: (1) reduce volume of Heart Manual text to be reduced for the Bangla patient audience, (2) develop a shorter ‘easy to use’ version for the Bangla Heart Manual; (3) develop an animated version of exercise programme; (4) develop an audio-visual version for relaxation and doctor-patient conversations, and (5) combine the two UK original Heart Manuals (for post myocardial infraction and revascularisation patients) into a single Bangla Heart manual. These identified areas informed the interview guides for the consultation meetings and workshops.

### Expert group consultation meeting and consultative workshop

A total of 28 participants attended the expert group meeting and consultative workshop (see Table 1). Two key themes were identified: (1) the sociocultural, physical and mental health context (2) current CR practice in Bangladesh (see Table 2). The implications of these themes on the adaptation of the Heart Manual materials are detailed in (see Table 3). It was agreed that the holistic CR approach (i.e. combination of exercise promotion, psychological support, and risk factor education) of the original UK Heart Manual intervention was an appropriate model for Bangladesh CR practice. How the specific elements of the UK Heart Manual were adapted are detailed in the sections below.

**Table 1:** Summary of expert meeting and consultative workshop participants

Participant characteristics	Number (*N* = 28)
**Gender** Men	15
**Role**	
Cardiologist (consultant)	5
Cardiac surgeon (consultant)	1
Psychiatrist (consultant)	1
Public health expert	1
Medical officer (Cardiology and cardiac surgery)	4
Physiotherapist	5
Nutritionist (Dietician)	2
Nurse	3
Patient counsellor	1
Medical Technologist	1
Patient	2
Caregiver	2
Total	28

**Table 2:** Key themes and their implications for adaptation of Heart Manual delivery

Theme	Analytical observation	Implication for adaptation	Adaptation of intervention delivery
Sociocultural, physical, and mental health context	Communication and conversational language about rehabilitation	Healthcare professionals across healthcare settings need to have a day to day communication language to discuss rehabilitation interventions.	Adaptation should better use of conversational language.
	Initiation of CR (timing)	Timing of referral and uptake of CR need to be considered.	The adaptation of CR needs to be introduced from the beginning of the patient hospital admission and delivered before discharge from the hospital.
	Involvement of patients and caregivers	Family members and caregivers in Bangladesh are mostly responsible for psychological, financial, and social support.	Families and caregivers should be involved during the delivery of the adapted intervention.
Current practice	Lack of existing CR infrastructure	Need to develop staffing and infrastructure to support and develop CR services in Bangladesh.	Importance of adapting existing Heart Manual training to meet the needs of Bangladeshi healthcare professions who will deliver Bangla Heart Manual.
	Barriers for CR referral	Address the barriers to referral and need to encourage cardiologists and cardiac surgeons to refer patients to CR.	Involvement and cooperation with physicians in the adaptation process and establishment hospital CR referral systems.
	Regional and institutional variation of services	Majority of cardiac hospitals /services currently based in Dhaka.	Home-based programme well suited to a ‘de-centralised’ model of CR provision.

**Table 3:** Summary of the format, content, and materials for adaptation of the Bangla Heart Manual intervention

Topic	Intervention component	Adaptation of the intervention
Heart Manual approach	Cross-sectional approach	Facilitator training will have to encompass difficulties of cross-sectorial set-up.
	Role of healthcare professionals	Use same model as UK Heart Manual of health care professionals -as intervention facilitators – physiotherapists, nurses and other suitable professional groups.
	Target patient group	Need to meet the needs of patients with CHD (both post-MI and revascularisation) in Bangladesh.
Heart Manual content		Ensure that Bangla Heart Manual reflects the comprehensive nature of UK Heart Manual.
	Exercise training	Supplement manual with animated version and visual images to aid patients to follow the 6 weeks home-exercise programme
	Psychosocial management	Mental health content has been customised to meet the needs of the Bangladeshi people and adopted into Bangla
	Risk factor management	Adapt risk factor content to reflect Bangladeshi cultural setting e.g. change specific dietary examples.
Heart Manual materials & training	Heart Manuals for patients with CHD (post-MI and revascularisation)	Combine original post-MI and revascularisation UK Heart Manuals into one Bangla Heart Manual. Develop two versions of Bangla Heart manual: (1) longer version - provide ‘encyclopaedia’ for both facilitators and patients, (2) short version –more pictorial to assist patients to use.
	Weekly exercise, walking and activities records	Inbuilt progress tracking chart into the adapted Bangla heart manual.
	Facilitator documents and training	The facilitator training was conducted a hybrid format with an in-person session led by a Bangladeshi specialist adapted from a 2-day UK web-based course. This approach ensured local adaptation while maintaining the original training structure.

* Cross sectional approach-A type of research study in which a group of people is observed, or certain information is collected, at a single point in time or over a short period of time

## Bangla heart manual

### Patient manual

Whilst the UK Heart Manual was designed as a reference resource for patients, a consistent element of stakeholder feedback was the ‘overwhelming’ nature of the UK Heart Manual in terms of the volume of text and page length of manuals. As a result, it was unanimously agreed that the Bangla adapted version should comprise two versions - a full manual version that could be used by patients and caregivers as a detailed reference resource and a shorter pictorial version for easy day to day patient access/use. For clearer understanding, the adapted Bangla Heart Manual was split in 4 parts and reduce the text volume but in the context of content it was suggested to retain the core components of UK Heart Manual.

The original UK Heart Manual consisted of 3 parts (see e-Appendix 4).

Part-1: Your heart condition: the facts,

Part-2: The weekly programme (6 weeks tailored walking and exercise programme, stress management programme.

Part-3: Facts and advice to help your recovery.

The Bangla Heart Manual was adapted to consist of 4 parts comparison with original UK heart manual.

(see e-Appendix 5):

Part 1: How to use the Bangla Heart Manual.

Part 2: Weekly programme (6 weeks exercise programme and stress and mental health self-management).

Part 3: Information and advice to help you.

Part 4: Risk factors of heart disease.

The adapted Bangla Heart Manual set a 6-week home exercise programme after revascularisation where the original HM had exercise including stretching, muscles strengthening exercise. Patients, caregivers, and health care professionals suggested the need to demonstrate all the animated version of the 6 weeks home-exercise programme to the patients and their caregivers before discharge from hospitals. For delivery of CR in Bangladesh it is proposed that: (1) physicians (cardiologists) should consistently refer their patients for CR, (2) physiotherapists, nurses, medical technologists, dietitians, and patient counsellor would work on their specific roles as team members and (3) the physiotherapists and cardiac nurses having the primary responsibility to take forward CR and the Bangla Heart Manual with patients and their families.

### Recognise the needs of families and caregivers in Bangla heart manual

The involvement of families and caregivers is already integrated in the original UK Heart Manual. The adapted Bangla Heart Manual gives particular emphasis to the integration of the involvement of families and caregivers. The nature of the Bangladeshi population’s caregiving primarily involves their family members (e.g. spouses, children and other close relatives). Healthcare professionals considered the involvement of caregivers as a key potential aspect of CR. Family members highlighted some topics as particularly relevant to their needs, i.e. handling ‘cardiac emergencies’, managing patient’s ‘smoking cessations’, and ‘stress management’. The Bangla Heart Manual Family Resource therefore seeks to emphasise self-care activities to support the mental and social well-being and risk factors of caregivers.

### Progress tracking by keeping of weekly exercise, walking and activities records

The progress tracker is a resource to enable patients to keep a record of their participation in the Heart Manual programme. The tracker was widely accepted by the stakeholder group in its original format and, it was considered readily adaptable by simply translating its content.

### Facilitator training course material

The two-day web-based Heart Manual facilitator training course delivered in English by the UK team at Lothian Health Board was considered appropriate to the Bangladeshi setting. However, it was also agreed that this UK training needed to be supplemented with a face-to-face Bangladeshi training session lead by a certified Heart Manual facilitator and experienced CR practitioner. This additional session would particularly highlight the differences between UK and the adapted Bangla Heart Manual materials. The original UK and Adapted Bangla Heart Manual materials (see e-Appendix 6).

### Finalisation of the Bangla heart manual

The amended Bangla Heart Manual materials underwent a final stage of professional proof reading and printing (Front covers shown in e-Appendix 7).

## Discussion

Despite the advancement and accessibility of cardiac care technology, including cardiac revascularisation and medical treatment, there has been less focus on the provision of CHD secondary prevention and rehabilitation services in Bangladesh [19, 23]. Home-based programmes offer the opportunity to improve access to CR. In this paper we describe the process for adapting the Heart Manual home-based CR programme (originally developed and evaluated in the UK) for patients with CHD and their caregivers based in Bangladesh - the ‘Bangla Heart Manual’.

Our evidence-based process of adaptation and stakeholder (patients, caregivers, clinicians, and service providers) involvement identified several key areas for the adaptation the original UK Heart Manual into the Bangladeshi healthcare system. First, we identified the lack of current health care professionals’ knowledge and skills in CR, emphasising the need for training and capacity building. Second, the UK manual content was considered too dense, text-based, and long. The adapted Bangla Heart Manual therefore focused on less text and more pictorial format but keeps the core components of CR of the original intervention. The Bangla Heart Manual incorporates two main adaptations: (1) the addition of a short manual ‘easy to use’ version for patients using and (2) development of an animated video version of the exercise programme available for smart mobile or computer/ laptop. The Bangla Heart Manual caregiver resource was adapted to reflect the strong tradition and culture of family involvement and responsibility in healthcare provision in Bangladesh [24]. This included maximising the opportunity to improve the health outcomes of family members and friends who may face the burden of supportive care for those with CHD,

We believe this to be the first study to describe the process of formally adapting a CR intervention in a LMIC. Eghøj and colleagues recently published their adaptation of home-based CR programme (REACH-HF) originally developed in UK for patients with heart failure and their caregivers in the Danish healthcare setting [25]. Our recent systematic scoping review reported healthcare intervention adaptation studies focusing on LMICs [26]. This scoping review showed, In the context of underdeveloped nations, a limited number of studies have detailed the modifications made to various cardiovascular disease therapies [26–28]. Implementing existing evidence-based interventions in new contexts offers a more efficient approach than developing new interventions for every geographical context. The clinical and cost effectiveness of the UK Heart Manual home-based intervention is supported by randomised trials in patients following myocardial infarction and revascularisation [29, 30]. A recently published a single centre quasi-randomsied trial comparing home-based CR plus usual care versus usual care alone in Bangladesh, showed patients outcome benefits with CR participants. This study provides proof of concept of the feasibility and acceptability of a home-based CR approach in this setting [31].

A key strength of this study was the application of a formal methodological process for intervention adaptation [20, 22]. However, we acknowledge there are limitations. First, while we sought to involve a diverse population of stakeholders, we cannot claim this to be a representative sample. We purposively sampled stakeholders from different specialised cardiac hospitals and academic institutions in Bangladesh. Second, this study focuses on the adaptation process. A pilot trial is planned to evaluate the feasibility and acceptability of the adapted Bangla Heart Manual for Bangladeshi healthcare professionals, patients, and their caregivers to inform future national implementation potentially wider deployment in the future.

Using an evidence-based approach, the UK Heart Manual programme was contextually and culturally adapted for people with CHD in the Bangladesh healthcare setting. The Bangla Heart Manual provides the opportunity for access to home-based CR in a low resource setting. Further research is needed to confirm the feasibility and acceptability of the delivery of the adapted Bangla Heart Manual for healthcare practitioners, patients, and their families.

## Conclusion

Adapting interventions with an existing evidence base, offers healthcare systems a more efficient approach to implementation than the development of a new intervention. Following an evidence-based approach, we adapted the UK Heart Manual home-based CR intervention to the Bangladesh healthcare context. Although this adaptation maintained the core components of the original intervention, the adapted Bangla Heart Manual included the development of a shorter ‘easy to use’ version of the manual and an animated version of the 6-week exercise programme. The feasibility and acceptability of the adapted Bangla Heart Manual programme for patients, caregivers, healthcare providers, and service providers will be tested in future pilot trial study in Bangladesh.

## Supplementary Information

Below is the link to the electronic supplementary material.

Supplementary Material 1

Supplementary Material 2

## Data Availability

The datasets generated for this study are available on email request to the corresponding author.

## Abbreviations

CHD: Coronary heart disease
CR: Cardiac rehabilitation
HM: Heart manual
LMICs: Low-and middle-income countries
REACH: HF-Rehabilitation Enablement in Chronic Heart Failure
