# Supplementary material for: Adaptation of an evidence-based home cardiac rehabilitation programme for people with coronary heart disease in Bangladesh
Source: BMC Health Serv Res. 2026 Mar 20;26:837. doi: 10.1186/s12913-026-14056-6 (PMC13270584; doi:10.1186/s12913-026-14056-6)
Supplement: Supplementary file 1 — Supplementary Material 1 [file 12913_2026_14056_MOESM1_ESM.docx]

**e-Appendix 1: Details of the seven-phase procedure.**

***Phase 1:* Select evidence-based intervention**

We selected ‘The Heart Manual’ intervention, originally designed and evaluated in the UK given its very widespread used and citation in over 500 peer-reviewed publications. ^26^ It is an individually tailored 6-week home-based CR programme for patients recovering from acute MI and/or revascularisation and is available in either paper or digital format. Founded on cognitive behavioural principles, it provides patients with a tailored approach to promote their self-management and well-being. ^27^ As a trained healthcare professional guided programme, the Heart Manual addresses cardiac misconceptions and enables individuals to use effective coping strategies and techniques throughout their recovery. The health professional visits the patient (and their caregiver) at their home or alternative suitable venue and introduces the manual. This is followed by 5-10 minute telephone calls/home visits at weeks 2, 3, 4, and 6 to check the patient's progress. The final contact at week 6 is used to agree on future self-management goals going forward. The Heart Manual programme is summarized using the TIDieR (Template for Intervention Description and Replication) checklist in (e-Table 3). ^28, 29^

***Phase 2:*** ***Review of the UK Heart Manual (phase 2) with intervention developers***

Hard copies of UK Heart Manual with relaxation CD materials were obtained and a web-meeting held with the developers to determine the core intervention components of the intervention. One member of the study team (JU) attended the online two-day UK Heart Manual facilitator training event (August 17th-18th, 2023) to clarify the intervention delivery and check context fit.

***Phase 3:*** ***Translation of intervention materials***

The Heart Manual was translated from English into Bangla by a translator. A backward translation was checked for translation accuracy by a second translator.

***Phase 4: Map similarity and difference between original and new contexts and identification of intervention adaptions***

We checked the potential mismatch and differences between the original and adapted settings of UK and Bangladesh including the intervention population and health setting (see e-Table 4). The expert group consultation meeting was held on 10^th^ October’2023 and included a cardiologist, cardiac surgeon, senior physiotherapist, senior nutritionist, senior staff nurses, public health expert. Following this meeting, the ‘Bangla Heart Manual’ version was drafted. -In addition to that,, one consultative workshop was organised (10^th^ January 2024) including the relevant professional experts i.e. cardiology medical officer, cardiac physiotherapists, cardiac staff nurses, nutritionist who are the service providers and with patients and their caregivers to adapt and check the Bangla Heart Manual understandability and deliverability.

**Phase 5: Summarizing feedback from consultation meeting and workshop for further revision.**

The draft Bangla Heart Manual materials were further reviewed by the expert group. This group identified intervention elements that might need adaptation to the context of resources and structure limitations of the Bangladesh health care system and the needs of patients and their caregivers. Feedback from the expert group consultation meeting and workshop were summarized, combined and used to inform a further round of Bangla Heart Manual revision.

**Phases 6 and 7: External review and finalisation of adapted intervention.**

The draft Bangla Heart Manual and the feedback from both expert group consultation meeting and consultative workshop were brought together and the final version of the Bangla Manual produced.

**e-Appendix 2: Adaptation of the Bangla Heart Manual semi structured questionnaire**

| **S/L** | **Open-ended questionnaire Items** | **Your opinions/suggestions** |
| --- | --- | --- |
| 01. | **Content of the ‘Bangla Heart Manual’.**   - Readability - Consistency - Navigability |  |
| 02. | **Language /pictures understand ability.**   - Structural proficiency - Understand ability according to context. - Visual clearance |  |
| 03. | **Bangla Heart Manual-type of materials**   - One original reference book - One mini version booklet - 6 weeks home-based exercise program with animated video - Relaxation audio and video version |  |
| 04. | **Mode of delivery:**   - Hybrid -centre based and - Weekly phone call |  |
| 05 | **Role of patients and caregivers to increase the acceptability of this Heart Manual** |  |

**e-Appendix 3: Expert group consultation meeting and consultative workshop for adaptation of the Bangla Heart Manual.**

| **A.** | **Methods of adaptation process:**   1. Selection of the eligible participants, 2) Invitation for a prescheduled meeting and 3) Provide Bangla Heart Manual to the stakeholders prior to the meeting. | | |
| --- | --- | --- | --- |
|  | **Expert group Meeting and consultative workshop** | **Stakeholders** | **Questionnaire** |
| B. | **International expert group consultation meeting ‘Heart Manual’ UK expertise and Bangladeshi expertise** | | |
|  | **International consensus meeting.**  (Via Zoom) | Three experts from UK and three from Bangladesh | Discussed content rearrangement of the original “Heart Manual” ) and combined both MI and revascularization heart manual and making it a single manual as CHD ‘ Bangla Heart Manual. We discussed the service provider and the mode of delivery of the intervention. |
| C. | **Expert group consultation meeting in Bangladesh (Held on 10^th^ October’2023)** | | |
|  | **Expert group consultation meeting** | 1. Consultant Cardiologist (ICHRI, BSMMU; NHFH&RI, NICVD) 2. Cardiac Surgeon (From ICHRI) 3. Psychiatrist ( from National Institute of Mental Health) 4. Public Health Specialist (from BUHS) 5. Senior Physiotherapist (from ICHRI, BSMMU and NHFH&RI) 6. Senior staff nurse ( from ICHRI, BSMMU and NHFH&RI). 7. Senior Nutritionist (from ICHRI, BSMMU and NHFH&RI). 8. Academician of rehabilitation science ( Bangladesh open university and Jessore University of Science & Technology). | **Open-ended questionnaire:**   1. Contents of ‘ Bangla Heart Manual’ 2. Sequence of materials of contents 3. Language /pictures understandability 4. Service providers (Who would be the potential CR service provider) 5. Mode of delivery (Center based or Hybrid -Centre   and centre monitored home based via Phone call) |
| D | **Consultative workshop in Bangladesh (Held on 10^th^ January’2024** | | |
|  | **Consultative workshop** | 1. Medical officer (Cardiology and cardiac surgery department of ICHRI) 2. Physiotherapist ( From ICHRI and BSMMU) 3. Nutritionist (From ICHRI) 4. Staff nurse (From ICHRI and BSMMU) 5. Medical technologist (From ICHRI) 6. Patient counselor (From ICHRI). 7. Patients and patients caregivers (From ICHRI and BSMMU). | **Open ended questionnaire:**  1. **Content of the ‘ Bangla Heart Manual’.**   - Readability - Consistency - Navigability   **2.** **Language /pictures understand ability.**   - Structural proficiency - Understand ability according to context. - Visual clearance   3. **Bangla Heart Manual-type of materials**   - One original reference book - One mini version booklet - Animated 6 weeks home-based exercise - Relaxation audio-visual version   4. **Mode of delivery:**   - Hybrid -centre based and Weekly phone call  1. **Role of patients and caregivers to increase the acceptability of this Heart Manual** |
| E. | **Record keeping:** By Tap recorder and manual note keeping | | |
|  | Finalize and summarized by making meeting minutes | | |

**e-Appendix 4: Description of original UK Heart Manual’ programme based on the TIDieR checklist**

| **NO.** | **Item** | | **Item description of ‘Heart Manual’** |
| --- | --- | --- | --- |
| 1. | Brief Name | Provide the name or a phrase that describes the intervention | **‘The Heart Manual’** intervention for patients with MI and revascularization |
| 2. | Why | Describe any rationale, theory, or goal of the elements essential to the intervention | The ’Heart Manual’ is designed to guide the patient and (their) family caregivers to use for a six-week home-based CR programme consisting of written materials, sections to record progress, a Q&A audio and relaxation programme facilitated by a specially trained clinician. The ‘Heart Manual’ not only focuses on short-term recovery but aims to assist the patient to adopt and maintain self-management skills, promoting the maintenance of well- being from their event or intervention on to long-term self-management. It also contains all the elements of a comprehensive rehabilitation programme: Health behaviour change and education, lifestyle risk management, exercise, medical risk management and psychological health.  National Institute for Health and Care Excellence (NICE) has identified the Heart Manual as a comprehensive, validated home-based cardiac rehabilitation programme in its clinical guidelines for secondary prevention since 2007. ^36^  Robust evidence found in a different systematic review, the home-based programmes using the Heart Manual were as equally effective as hospital-based rehabilitation. ^37^ It also concluded that the Heart Manual is as effective as hospital-based cardiovascular rehabilitation on a number of psychological, behavioural, biological, service and cost outcomes. ^38^ The Heart Manual included in a review of home-based and recently developed telehealth programmes, was identified as an effective alternative to hospital-based programmes. ^39^ |
| 3. | What | **Materials:** Describe any physical or informational materials used in the intervention, including those provided to participants or used in intervention delivery or in training of intervention providers. Provide information on where the materials can be accessed (such as online appendix, URL) | The Heart Manual intervention package is written in simple language and laid out in **three, easy-to-follow parts**:  **Part 1: Your Heart Condition:** **The Facts.** This part contains important information for the patient to read during the initial phase of their recovery.  **Part 2: The Weekly Programme**: This part consists of six weekly sections, each containing important information to aid the patient’s recovery, a graded exercise programme and a focus each week on a lifestyle risk factor.  **Part 3: Facts and Advice to Help Your Recovery**: This part contains important information about the patient’s recovery, medication, and other significant issues relating to their condition such as hospital investigations and treatments. |
| 4. |  | **Procedures:** Describe each of the procedures, activities, and/or processes used in the intervention, including any enabling or support activities | The key components and activities for a comprehensive cardiac rehabilitation programme are included in the Heart Manual supported by a specially trained clinician:   - An exercise programme - Lifestyle and risk factor education - Advice about safe and unsafe activities including pacing - The common psychological responses and their management - Stress management and relaxation Information about frequently prescribed medications - Event and intervention specific information, investigations and treatments.   Patients should be encouraged to use the Heart Manual daily to help identify, plan and monitor weekly targets and pacing activities. Psychological support and health behavior change activities are imbedded throughout the manual. |
| 5. | Who provided | For each category of intervention provider (such as psychologist, nursing assistant), describe their expertise, background, and any specific training given | Patients should be assessed by a registered practitioner (nurse or AHP) as suitable to commence the programme. Non-registered/ non-regulated health care workers such as exercise specialist or health care support workers may be trained in the resources and deliver aspects of the programme under the supervision of a registered practitioner according to each individuals clinical competence. |
| 6. | How provided | Describe the modes of delivery (such as face to face or by some other mechanism, such as internet or telephone) of the intervention and whether it was provided individually or in a group | **In hospital:** The ‘Heart Manual’ first given in the Hospital through face-to-face communication. Initial in hospital approach for recruitment-referral made /cardiac rehab (CR) team visit.  **Other form**-CR practitioner picks up referral and approach to made first contact with the patients by physically or phone for initial assessment confirmation and then send ‘Heart Manual’ to patient.  **Consultation format:** Formal assessment and consultation, information given/objective settings in clinic/ home/ or by phone/video call.  **Follow up:** Phone follow-up, e.g., week 2/4. Tailored lifestyle content, objective and behaviour change by Phone/Video call.  **Final consultation check:** Understanding, long term plan, emergency information by clinic/ home/ or by phone/video call. |
| 7. | Where | Describe the type(s) of location(s) where the intervention occurred, including any necessary infrastructure or relevant features | **Its flexibility:**   1. In hospital/ Clinic 2. Home or alternative/suitable venue-e.g. community centre/hall 3. Phone/ video call |
| 8. | When and how much | Describe the number of times the intervention was delivered and over what period of time including the number of sessions, their schedule, and their duration, intensity, or dose | **Initial assessment consultation will take about 1 hour.**   - Subsequent contacts are likely to average about 30-45 mins. - **Recovery program:** Following 6-week recovery programme:   **Week-1 Program:**   - Answer some more questions about your condition - Introduce you to the Exercise and Relaxation Plans which will play an important part in your recovery and afterwards. - Look at some of the things which might be worrying you, and show you how you can fight back. - If you live with someone, get them to read this section - it can help them as much as it helps you.   **Week-2 program:**   - Try to carry on with the Exercise/Activity Plan. - Don't forget to fill in your walking, exercise and activity record sheets. If you do this, you'll be able to look back - at them in a few weeks and see how much you have improved. - You will keep listening to the relaxation audio and get some tips about what to do if you are worried. - You will learn about your coronary artery disease and the risk factors that contributed to it. - You will find out what happened to John McKay (case study). - You will read about the biggest risk factor of all - smoking.   **Week-3 program:**   - Keep on building up your Exercise/Activity Plan. If you didn't start regular walking last week, try to do so now, as advised. If you did start regular walking, you should be building up the distance. - Don't forget to fill in your walking, exercise and activity record sheets. - Try to keep listening to the relaxation audio and get some information about stress and how to control it - This week's risk factor is diet. Most people find that healthy food is also tasty - try it!   **Week-4 program:**   - Keep building up your Exercise/Activity Plan. You should also be walking daily and increasing the frequency, speed and distance as advised. - Remember to fill in your walking, exercise and activity record sheets. - Keep listening to the relaxation audio. - There’s more this week about stress. A stressful lifestyle can play a big part in building up risk factors so it’s helpful to learn ways to manage it. - This week’s risk factor is being overweight – a major problem for some people. But getting control of your weight can have a big benefit in reducing the risk of further heart problems in the future.   **Week-5 program:**   - You'll keep building up your Exercise/Activity Plan. - Remember to fill in your walking, exercise and activity record sheets. - Keep listening to the relaxation audio. - You'll hear more about stress. If you are thinking more and more about diving back into a busy lifestyle, you'll find some very useful advice about speeding, overworking and driven behaviour. - This week's risk factor is lack of exercise. If you have been following the Exercise/Activity Plan you should already be seeing benefits from regular exercise, but this section goes into a little more detail about why it's so important. - There's a section about sex (if you're interested).   **Week 6 - the last week of the Manual program:**   - You'll keep building up your Exercise/Activity Plan. - Remember to fill in your walking, exercise and activity record sheets. - Keep listening to the relaxation audio. - We'll be looking at hostility and anger. - We will think about going back to work. You may wish to discuss this with your GP or facilitator. - This week's risk factor is high blood pressure. - We shall bring together the different aspects of the stress control programs - We shall review what has happened over the last 6 weeks and provide a checklist for future actions. - Contacts can be face to face at home or in clinic, by telephone or video call (first contact benefits from being face to face if service provision allows) - Phone calls are useful for checking in on patients between longer consultations If visiting patients at home, allow time for travel, record keeping etc. |
| 9. | Tailoring | If the intervention was planned to be personalised, titrated or adapted, then describe what, why, when, and how | Be flexible:   - shorter, more frequent consultations may be useful for some patients - some patients will need minimal input, shorter and/ or fewer sessions |

**e-Appendix 5: Comparison between the original and the modified intervention with rational for adaptations of the Heart Manual (HM) for home-based CR of Bangladeshi patients with CHD**

| **S/N** | **Heart Manual content themes** | **Original Heart Manual, UK (Three parts)** | **Adaptation-Bangla Heart Manual - Bangladesh (Four parts)** |  |
| --- | --- | --- | --- | --- |
|  | **Part of Heart Manual** | | | |
| **1.(A)** | **Part-1: Section A: Your Heart Attack- the Facts:** | This section is for you to read while you are recovering from heart attack- It tells you the plain facts about your heart condition after procedures. Focused on:   - Discuss about heart and coronary heart disease - Intervention procedures (Angioplasty and CABG).   Some general tips: What to do and what not to do after procedure | **Part-1: Rules of using the Bangla Heart Manual**  (How to use the Cardiac Rehabilitation  Programme book?)   - Discuss about heart and coronary heart disease - Intervention procedures (Angioplasty and CABG). - Some general tips: What to do and what not to do after procedure (During recovery phase after procedures). |  |
| **1.(B)** | **Part 2: The Weekly Programme and risk factors of CHD and its management** | This part consists of six weekly sections: Each weekly section has important to help you to recover, and an exercise programme to help you back to fitness in easy stages.   - Follow your plan morning and evening as advised by your facilitator - Each time marks your activities in the chart provided   Practice relaxation by listening to tracks 1 and 2 on the relaxations CD or audio. | **Part-2: Weekly exercise programme and stress management through relaxation.**   - Wach week formative home exercise programme (week 1-6) - Record keeping or mark your activities in the chart provided of walking, exercise and daily activities records. - Stress management and relaxation practice (by using relaxation audiovisual version) |  |
| **1.(C)** | **Part 3: Facts and Advice to Help Your Recovery.** | This part provides extra information about your recovery, medications and other things you might want to know.   - Wound healing. - Swollen hands or feet. - Reasons for using drugs. - Some routine tests in the hospital. - Why does angina or chest pain occur? - Other chest pains. - Shortness of breath. - Hyperventilation. - Chest palpitations - Depression following procedures. - Sleep problems and so on. | **Part-3: Information and advice to help you**   - Wound healing. - Swollen hands or feet. - Reasons for using drugs. - Some routine tests in the hospital. - Why does angina or chest pain occur? - Other chest pains. - Shortness of breath. - Hyperventilation. - Chest palpitations - Depression following procedures. - Sleep problems. - Some practical examples of mental problems and overcoming these conditions |  |
| **1.(D)** | **Don’t have part-4 (Original UK Heart Manual)** | **Risk factor’s part has been laps with (part 2) the weekly programme section.** | **Part-4: Risk factors of CHD and its management. (Make an individual part as part 4)** |  |
| **2.** | **Mode of delivery** | | | |
|  | Home visit | Home visit (Nurses/physiotherapist) | No home visit (Phone/mobile call from institute) |  |
|  | Hospital setting | Hospital setting cardiac rehab intervention facilities | Hospital setting (Monitored-intervention delivered before or during discharge) |  |
|  | Telephone call | Phone call from hospital | Weekly phone call from hospital |  |
| **3.** | **Responsible personnel (Health Care Staff)** | | | |
|  | Nurses driven | Nurse driven (Cardiac rehab team) Team consist of:   - Cardiologist - Staff CR nurse - Physiotherapist/exercise physiologist - Occupational therapist - Nutritionist - Patient counselor | Physiotherapist driven (Cardiac rehab team)  Team consist of:   - Cardiologist - Physiotherapist (Cardiac rehab expert) - Cardiac staff nurse - Medical technologist - Nutritionist |  |

**e-Appendix 6: The original UK and Adapted Bangla Heart Manual materials (Supplementary illustrations)**

**Front covers of the original Heart Manual Front covers of the adapted Bangla Heart Manual**
