# Supplementary material for: Adaptation of an evidence-based home cardiac rehabilitation programme for people with coronary heart disease in Bangladesh
Source: BMC Health Serv Res. 2026 Mar 20;26:837. doi: 10.1186/s12913-026-14056-6 (PMC13270584; doi:10.1186/s12913-026-14056-6)
Supplement: Supplementary file 2 — Supplementary Material 2 [file 12913_2026_14056_MOESM2_ESM.docx]

**e-Table 1: Potential matches and mismatches with original and adapted intervention and population.**

| **Adaptation characteristics** | **Original UK Heart Manual** | **Bangla Heart Manual,** |
| --- | --- | --- |
| **Population characteristics:** |  |  |
| Language | English | Bangla |
| Ethnicity | Diverse (White British, European, African etc..) | Primarily Bengali |
| Economic status & funding | Funded by the public National Health Service (NHS) | Mostly, patient out of pocket expenditure) |
| Educational level | The literacy rate for adults (15+) in the UK is 99% | The literacy rate for adults (15+) in Bangladesh is 75.6% |
| Major CHD risk factors (Chronologically): | 1. High BP; 2. high blood cholesterol levels; 3. smoking; 4. diabetes; 5. overweight or obesity; 6. lack of physical activity; 7. unhealthy diet and stress | 1. Hyperlipidemia (83%) 2. Physical inactivity (51%) 3. Obesity (46%) 4. Smoking (44%) 5. Family history of CAD (22%) 6. Diabetes (15%) 7. Unhealthy diet. |
| **Intervention delivery characteristics** | | |
| Healthcare staff | Great access to experienced and trained nurses and physiotherapists | Limited number of experienced of allied health care staff. |
| **Community context** |  |  |
| Transportation status | Usually good local and timely transportation access | Transportation system more limited and time consuming. |
| Family structure | Some patients are self-dependent and some are family dependent. | Most of them are family oriented and dependent on family. |
| Personal management culture | Most of them have their individual responsibility | Predominantly family responsibility culture. |
